# The reform study: a cohort multiple randomised controlled trial

**DOI:** 10.1186/1745-6215-14-S1-O44

**Published:** 2013-11-29

**Authors:** Sarah Cockayne, Joy Adamson, Catherine Hewitt, Robin Hull, Anne-Maree Keenan, Antony Redmond, Sallie Lamb, Caroline McIntosh, Hylton Menz, Wesley Vernon, Judith Watson, David Torgerson

**Affiliations:** 1 University of York, York, UK; 2 University of Leeds, Leeds, UK; 3 Harrogate & District NHS Foundation Trust, Harrogate, UK; 4 Sheffield Teaching Hospitals NHS Foundation Trust, Sheffield, UK; 5 La Trobe University, Melbourne, Australia; 6 NUI Galway, Galway, Ireland; 7 University of Warwick, Warwick, UK

## Background

Undertaking randomised controlled trials can be challenging. Many trials fail to recruit, there may be issues around consent, patient preferences and treatment comparisons. The patient preference and Zelen designs have attempted to address the issues around recruitment and patient preference. However the cohort multiple randomised controlled trial has been suggested as an alternative design. The REFORM study uses this design to evaluate the clinical and cost effectiveness of a multifaceted podiatry intervention consisting of an orthotic, foot and ankle exercises and footwear advice for the prevention of falls in the over 65.

## Methods

Community dwelling patients over the age of 65 who have attended a podiatry clinic will be sent an REFORM invitation pack. 1,700 participants will be recruited to the REFORM cohort all of whom will complete monthly falls calendars and questionnaires which include the EQ5D, activities of daily living and fear of falling at baseline, 6 and 12 months. Data on participants who decline to be in the cohort will be collected where possible. Ethics approval has been given to invite participants who agree to be contacted again about other podiatry studies. Once the cohort is assembled the eligible patients in the cohort will be identified and a random sample of 445 participants will be offered the package of podiatry care. We will compare the outcomes of those randomly selected to receive the intervention to those who were eligible but were not randomly selected to receive the intervention i.e. those receiving usual podiatry care.

